# Comparative Therapeutic Effects of Minocycline Treatment and Bone Marrow Mononuclear Cell Transplantation following Striatal Stroke

**DOI:** 10.1155/2017/1976191

**Published:** 2017-06-21

**Authors:** Celice C. Souza, Michelle Castro da Silva, Rosana Telma Lopes, Marcelo M. Cardoso, Lucas Lacerda de Souza, Adriano Guimarães Santos, Ijair Rogério dos Santos, Edna C. S. Franco, Walace Gomes-Leal

**Affiliations:** Laboratory of Experimental Neuroprotection and Neuroregeneration, Institute of Biological Sciences, Federal University of Pará, Belém, PA, Brazil

## Abstract

We explored the comparative effects of minocycline treatment and intrastriatal BMMC transplantation after experimental striatal stroke in adult rats. Male Wistar adult rats were divided as follows: saline-treated (*N* = 5), minocycline-treated (*N* = 5), and BMMC-transplanted (*N* = 5) animals. Animals received intrastriatal microinjections of 80 pmol of endothelin-1 (ET-1). Behavioral tests were performed at 1, 3, and 7 days postischemia. Animals were treated with minocycline (50 mg/kg, i.p.) or intrastriatal transplants of 106 BMMCs at 24 h postischemia. Animals were perfused at 7 days after ischemic induction. Coronal sections were stained with cresyl violet for gross histopathological analysis and immunolabeled for the identification of neuronal bodies (NeuN), activated microglia/macrophages (ED1), and apoptotic cells (active caspase-3). BMMC transplantation and minocycline reduced the number of ED1+ cells (*p* < 0.05, ANOVA-Tukey), but BMMC afforded better results. Both treatments afforded comparable levels of neuronal preservation compared to control (*p* > 0.05). BMMC transplantation induced a higher decrease in the number of apoptotic cells compared to control and minocycline treatment. Both therapeutic approaches improved functional recovery in ischemic animals. The results suggest that BMMC transplantation is more effective in modulating microglial activation and reducing apoptotic cell death than minocycline, although both treatments are equally efficacious on improving neuronal preservation.

## 1. Introduction

Acute neural disorders are untreatable conditions characterized by rapid cell death and tissue loss rendering inexorable functional deficits. This can be observed following stroke [1, 2], spinal cord injury (SCI), and brain trauma [3]. Following acute neural disorders, primary pathological events induce vascular damage with tissue necrosis and subsequent secondary pathological effects, including excitotoxicity, neuroinflammation, apoptosis, and oxidative stress [1, 2]. In the case of stroke, the initial infarct area expands with time invading the ischemic penumbra, which worsens the neurological outcome [1, 2].

Neuroinflammation is an important secondary event following stroke [4, 5]. Ischemic damage triggers an intense inflammatory response with both humoral and cellular components, which contribute to both detrimental and beneficial actions [4, 5]. In hours, several cytokines are released by neurons and glial cells [6], which increase the recruitment of neutrophils [7, 8], lymphocytes [9–12], and intense microglia/macrophage activation [13–15].

It has been established that microglial activation has a dual role after stroke, with both beneficial and detrimental actions [4]. Inhibition of microglial activation with minocycline decreases the infarct area in both the cortex and striatum following middle cerebral artery occlusion (MCAO) [13, 15, 16]. Significance of neuronal preservation has been obtained by inhibition of microglia with PJ34, a poly(ADP-ribose) polymerase inhibitor [17]. In addition, it has been reported that active caspases, including caspases 3 and 8, modulate microglia activation contributing to their neurotoxicity [18]. On the other hand, several papers show that microglia may be beneficial after stroke [19–22]. This neuroprotective effects involve engulfment of neutrophils [20], modulation of excessive inflammation [22], and release of growth factors [12].

Cell therapy is a promising therapeutic approach to mitigate damage and functional impairment following stroke and other acute and chronic neural disorders [23, 24]. Several types of stem cells have been used for transplantation after stroke and SCI, including human neural stem cells [25], embryonic stem cells [26], oligodendrocyte progenitor cells [27], induced pluripotent stem cells [28], and adult stem cells [15, 16, 29]. Bone marrow mononuclear cells (BMMCs) modulate the pathological environment, decreasing inflammation, increasing angiogenesis by release of growth factors, and anti-inflammatory cytokines, which induce considerable neuroprotection and functional recovery following stroke [15, 16, 29].

We have previously shown that both minocycline and intravenous transplantation of BMMCs induce neuroprotection following striatal stroke [16, 30]. Nevertheless, it has been shown that most of the intra-arterial or intravenously transplanted BMMCs preferentially go to peripheral organs including the lung, spleen, and bladder rather to the brain [31–33]. A small percentage of intravenously transplanted BMMCs gets into the ischemic brain and dies in few days after transplantation [34]. Studies using intracerebral transplant of BMMCs are needed for the sake of comparison, considering that the number of cells getting into the brain is higher than using intravascular methods. It is possible that the afforded neuroprotection may be higher using intravascular methods. Studies comparing the anti-inflammatory and neuroprotective effects of minocycline treatment and intrastriatally transplanted BMMCs are not available.

In this study, we induced focal striatal ischemia using the ET-1 model of stroke in order to compare the effects of minocycline treatment and BMMC transplantation on an infarct area, neuronal loss, microglia activation, apoptosis, and functional recovery in different survival times postinjury.

## 2. Material and Methods

### 2.1. Animals

Male adult Wistar rats (220–280 g) were obtained from the Federal University of Pará animal house. All animals were treated with food and water available ad libitum. All experiments were performed following the Principles of Laboratory Animal Care (NIH publication number 86-23, revised 1985) and European Commission Directive 86/609/EEC for animal with protocol approved by the Ethics Committee on Experimental Animals of the Federal University of Pará. We handled animals using humane principles, and every possible effort was accomplished to avoid animal suffering and distress.

### 2.2. Model of Experimental Stroke

The method of experimental stroke used microinjections of the vasoconstrictor peptide endothelin-1 (ET-1) (Sigma, Saint Louis, MO, USA), according to our previous publications [7, 16]. This experimental method of ischemic stroke has been validated by several publications by ours [7, 15, 16, 35–37] and other research groups [38–43]. Animals were anesthetized with ketamine hydrochloride (72 mg/kg, i.p.) and xylazine hydrochloride (9 mg/kg, i.p.) and held in a stereotaxic apparatus abolishment of their corneal and paw withdraw reflex. The animal temperature was measured using a rectal thermometer, and temperature was maintained during the experiment using a homoeothermic blanket unit. Further, 80 pmol of ET-1 (Sigma, Saint Louis, MO, USA) in 1 *μ*l of sterile saline were injected into the rat's striatum (*N* = 10) over a period of 2 min using a glass micropipette. The pipette was maintained in the striatum for 3 minutes before slow removal. The following stereotaxic coordinates were used in relation to the bregma: +0.7 mm lateral, +2.5 mm anterior, and 4.0 mm deep from the pial surface in the dorsoventral axis [44]. After stroke induction, animals were maintained in individual cages with free access to food and water for 7 days.

### 2.3. Experimental Design and Animal Groups

Animals were randomly divided in the following experimental groups: animals with striatal stroke and treated with sterile saline (G1, *N* = 5); animals with striatal stroke and treated (i.p.) with minocycline (G2, *N* = 5); and animals with striatal stroke and intrastriatally transplanted with BMMCs (G3, *N* = 5). 1 rat was used as a BMMC donor in individual experiments.

### 2.4. Minocycline Treatment

We have used minocycline to inhibit microglial activation over 7 days following experimental striatal stroke. First, G2 animals were injected (twice a day) with minocycline (Sigma, Saint Louis, MO, 50 mg/kg, i.p.) for two days. Treatment started at 2 hours after ET-1 microinjections. Subsequent doses of minocycline (25 mg/kg, i.p.) were administered once a day for the next 4 days, and animals were perfused at 7 days poststroke. This protocol is routinely used by our group [15, 16].

### 2.5. Isolation of Bone Marrow Mononuclear Cells

The protocol for isolation of BMMCs was previously published by our group [15, 16]. Bone marrow was extracted from the femoral and tibial bones. BMMCs were quantified using a Neubauer chamber to a final concentration 10^6^ cells/ml. Twenty-four hours after induction of experimental striatal stroke, animals were intrastriatally injected with 10^6^ BMMCs. Isolated BMMCs were then incubated in Hoechst solution (1 *μ*g/ml, Sigma, Saint Louis, MO, USA) for 25 min at 18°C. This procedure was used to visualize BMMCs in the ischemic striatum.

### 2.6. Behavioral Analysis

Behavioral analysis was performed by blinded investigators to the data. Animals were tested 1 day before and 1, 3, and 7 days after ischemia. The following tests were used:

1. The open-field test [45]: The open field test used a box with dimensions of 60 × 60 × 50 cm and containing 16 square subdivisions of equal sizes. Experiments were performed using three trials (5 min each). In these trials, animals were first placed at the center of the open field. The motor performance was recorded by a video camera (Sony, USA). The behavioral parameters included the number of occurrences of the exploratory behavior of standing up on the hind legs (rearing), body self-cleaning (grooming) latency (time taken to leave the starting point), and distance travelled in the open field.
2. The modified sticky-tape test: We used a protocol previously published [46]. A small nonremovable tape sleeve (3.0 × 1.0 cm) was placed around the animal's forepaw. The time spent by the animal dealing with this physical stimulus was recorded. Animals were pretrained 24 hours before ischemia (two times a day for 30 seconds) with a 1-hour interval between each test. After ischemia, the test was performed at 1, 3, and 7 days postischemia. Each test consisted of five trials in which the two best performances were considered for quantitative analysis. The left and right paw performances were calculated to assess sensorimotor performance [46].

### 2.7. Perfusion and Histological Processing

Animals were deeply anesthetized and transcardially perfused with heparinized 0.9% phosphate-buffered saline (PBS) followed by 4% paraformaldehyde at 7 days after ischemia. Brains were postfixed for 24 hours in the same fixative and cryoprotected in different gradients of sucrose-glycerol solutions over 7 days. The tissue was then frozen in Tissue Tek, and 30 *μ*m coronal sections were cut using a cryostat (Carl Zeiss Micron, Germany). Sections were mounted onto gelatinized slides and stored in a freezer at −20°C.

### 2.8. Gross Histopathological Analysis

Gross histopathology was assessed in sections stained with cresyl violet (Sigma, Saint Louis, MO, USA). The infarct area caused by ET-1 injection was recognized by the presence of colanyl blue, tissue pallor, and necrosis [7].

### 2.9. Antibodies and Immunolabeling Protocol

To immunolabel cell bodies of mature neurons [47], activated macrophage/microglia [48], and apoptotic cells [15], we used the following antibodies: mouse anti-NeuN (1 : 100; Temecula, CA), mouse anti-ED1 (1 : 200, Serotec, UK), and rabbit anti-active caspase-3 (1 : 250, Promega), respectively.

The protocol used for immunohistochemistry was described in our previous studies [15, 49]. In short, slide-mounted sections were removed from the freezer, kept in a heating oven at 37°C for 30 min, and rinsed once in 0.1 M PBS for 5 min. To improve labeling intensity, sections were pretreated in 0.2 M boric acid (pH 9.0) previously heated to 65°C for 25 min. This temperature was left constant over the pretreatment period. Sections were further allowed to cool for about 20 min and incubated under constant agitation in 1% hydrogen peroxide in methanol for 20 min. Sections were rinsed 3 times (5 min each) in 0.05% PBS/Tween (Sigma, Saint Louis, MO, USA) and incubated with normal serum (horse normal serum for ED1 and NeuN and goat normal serum for caspase-3) in PBS for 1 h. Without further rinsing, sections were then incubated with the primary antibody diluted in PBS for 24 h, rinsed in PBS/Tween solution for 5 min (3 times), and incubated with appropriate secondary antibody for 2 h. Both primary and secondary antibodies were incubated at room temperature (20°C). As a negative control, PBS, rather than the primary antibody, was used. Sections were rinsed again for 5 min (3 times) and incubated in an avidin-biotin-peroxidase complex (ABC Kit, Vector Laboratories) for 2 h. Sections were then rinsed 4 times (3 min each rinse) and DAB reacted according to a protocol published elsewhere [15]. After the DAB reaction, sections were rinsed 3 times (3 min each) in 0.1 M phosphate buffer, dehydrated using alcohols and xylene, and cover slipped. Some sections were also counterstained with cresyl violet.

### 2.10. Qualitative Analysis

All sections stained with the different histological methods were observed using a light microscopy. Illustrative images from the more representative fields were obtained using a digital camera (Moticam 2500) attached to the microscope (Nikon Eclipse 50i, Nikon, Tokyo, Japan).

### 2.11. Quantitative Analysis

Lesion area (mm^2^) was measured from the pictures of cresyl violet-stained sections for the different experimental groups (3 sections/animal/survival time) using the NIH's free software, Image J. Images containing the lesion area were digitized using a digital camera (Moticam 2500) attached to the microscope (Nikon Eclipse 50i, Nikon, Tokyo, Japan). The lesion area was recognized by intense tissue pallor and inflammatory infiltrated at and around the lesion center. We used one lesion area per section and three sections per animal (*N* = 5). Using a scale bar, Image J was set to measure lesion area. Values were averaged and stored to statistical analysis. This protocol was previously used by our group [15].

We counted the numbers of activated microglia/macrophages (ED1-1+ cells), apoptotic cells (active caspase-3+ cells), and mature neuronal bodies (NeuN+ cells) per field in coronal sections, using a square 0.25 mm-wide grid (objective 40x) in the eyepiece of a microscope. This grid corresponds to an area of 0.0625 mm^2^ using 40x objective. We counted 16 fields per section and 3 sections/animal (*n* = 5 animals/survival time). Fields were counted in the whole striatal area according to a protocol previously published by our group [16]. Counts were averaged and plotted in Cartesian coordinates.

### 2.12. Statistical Analysis

Descriptive statistics was performed for all counts, and averages, standard deviations, and standard errors were calculated for all counts. Comparisons between the groups were assessed by analysis of variance (ANOVA) with Tukey post hoc test. Statistical significance was accepted for *p* < 0.05. All statistical analyses were performed using the Software GraphPad Prism 5.0.

## 3. Results

### 3.1. Microinjections of ET-1 Caused Focal Striatal Damage and Acute Inflammatory Response, Which Were Reduced by Both BMMC Transplantation and Minocycline Treatment

**Figure 1:**
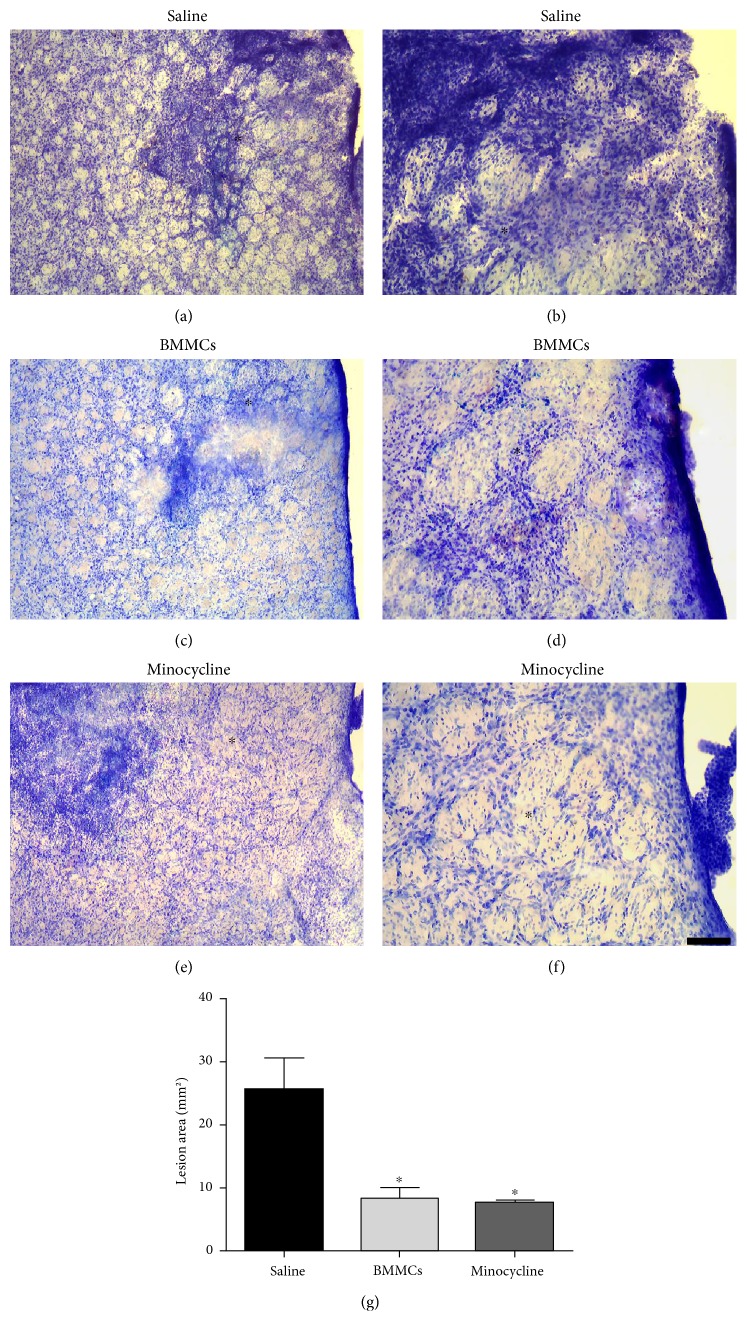
Histopathological analysis of the ischemic lesions stained with cresyl violet. Ischemic animals treated with saline solution (a and b), BMMCs (c and d), or minocycline (e and f). Quantitative analysis of the lesion area using Image J (g). ∗ demarcates the lesion center. Scale bars: 400 *μ*m (a, c, e); 40 *μ*m (b, d, f).

Microinjections of ET-1 into the rat striatum caused pallor, tissue loss, and intense inflammatory response, which is in agreement with previous reports (Figures 1(a) and 1(b)). Treatment with BMMCs (Figures 1(c) and 1(d)) or minocycline (Figures 1(e) and 1(f)) decreased inflammation and tissue loss in comparable proportions (Figure 1(g)). These results were confirmed by quantitative analysis (Figure 1(g)).

### 3.2. Transplantation of BMMCs after Focal Striatal Ischemia Reduces More Microglial Activation than Minocycline

**Figure 2:**
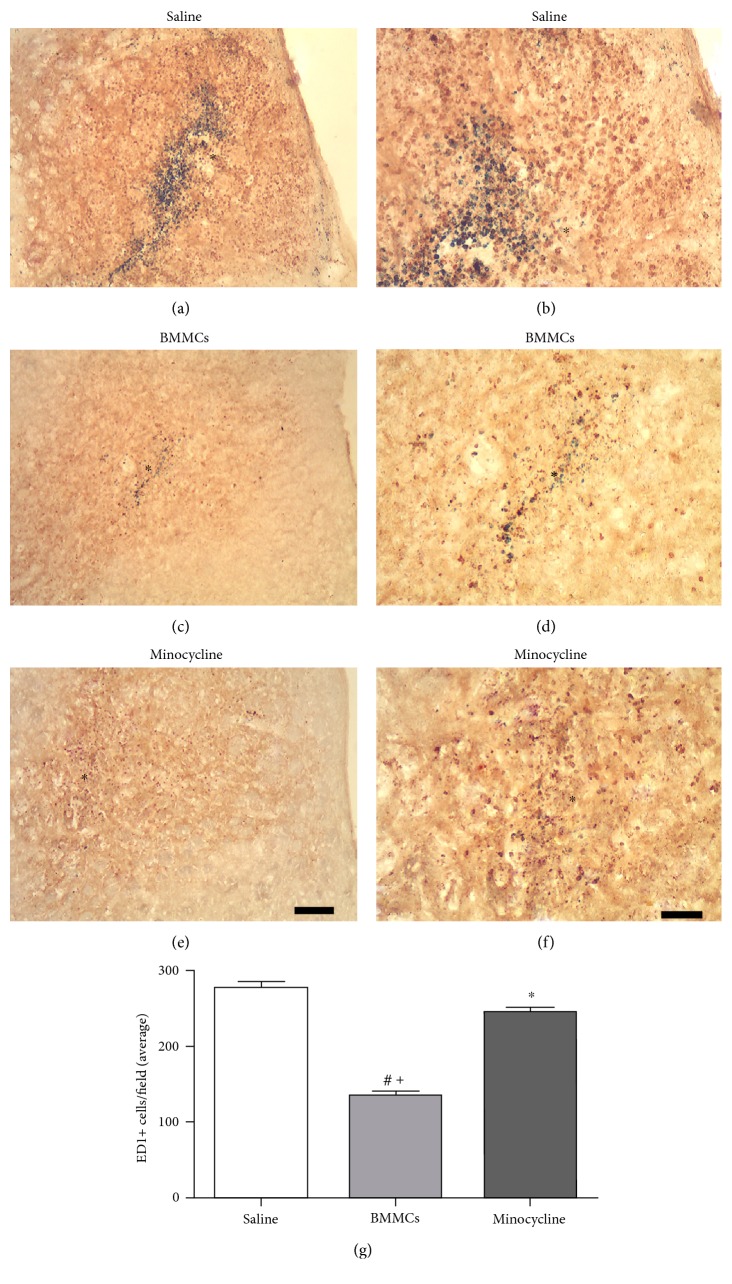
Inhibition of microglial activation by minocycline and BMMCs after striatal ischemia. Animals treated with saline (a, b), BMMCs (c, d), or minocycline (e, f) 7 days after ischemic induction. Quantification of ED1+ cells in all experimental groups (g). Minocycline treatment and BMMC transplantation reduced the number of ED1+ cells, with better results for BMMCs (*p* < 0.05).^∗^Comparison of saline versus minocycline. ^+^Comparison of saline versus BMMCs. ^#^Comparison of BMMCs versus minocycline. Arrows point to ED1+ cells. ∗ demarcates lesion center (a–f). Scale bars: 400 *μ*m (a, c, e); 40 *μ*m (b, d, f).

We have compared the effects of minocycline and intrastriatal BMMC transplantation following striatal ischemia on microglial activation. Both BMMC transplantation and minocycline treatment reduced the number of ED1+ cells in the striatum at 7 days, compared to that in saline-treated animals (Figure 2). Nevertheless, BMMC transplantation (Figures 2(c) and 2(d)) induced a higher microglial inhibition compared to minocycline treatment (Figures 2(e) and 2(f)). These results have been confirmed by quantitative analysis (Figure 2(g)). The averages for saline, BMMCs, and minocycline-treated animals were 276.3 (±9.3), 133.8 (±6.8), and 244.6 (± 7.1) [ED1+ cells/field (±SD)], respectively.

### 3.3. BMMC Transplantation and Minocycline Treatment Induce Comparable Neuroprotection following Focal Striatal Ischemia

**Figure 3:**
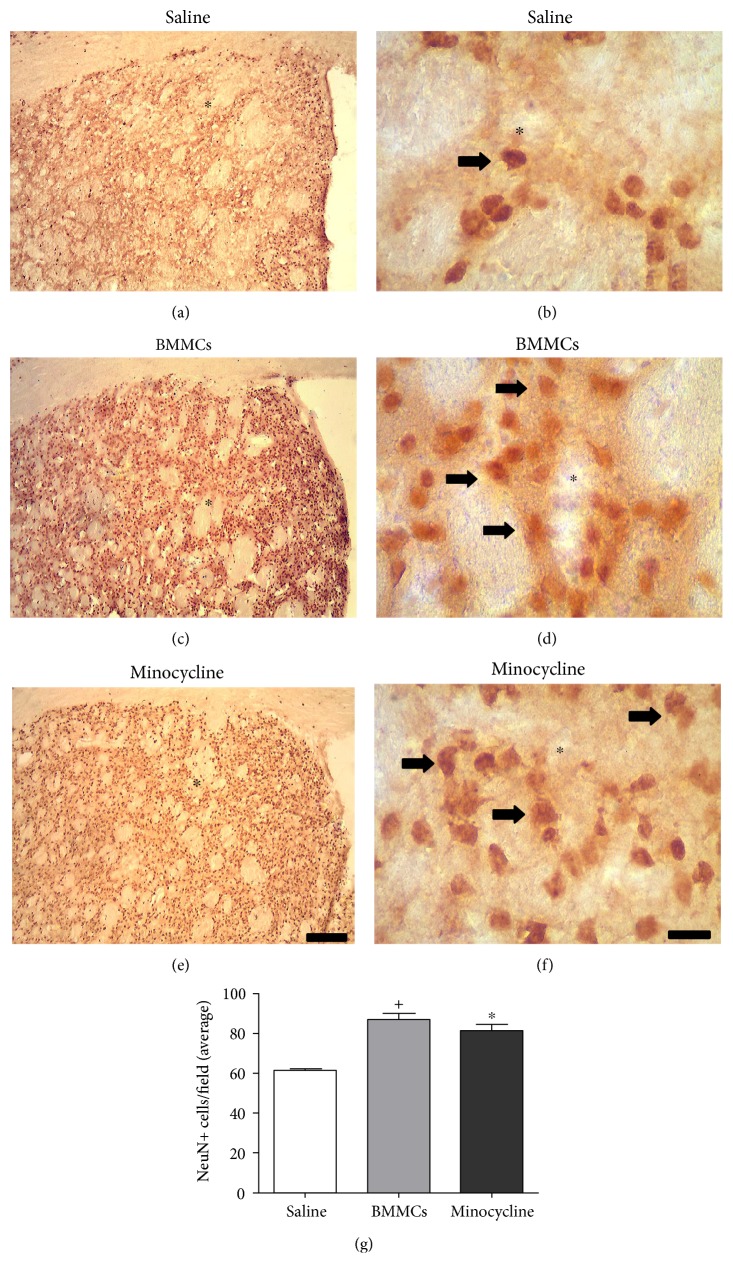
Neuroprotection by BMMCs and minocycline after striatal ischemia. Animals treated with sterile-saline (a, b), BMMCs (c, d), or minocycline (e, f) 7 days postischemic induction. Quantification of NeuN+ cells in all experimental groups (g). BMMCs and minocycline treatments significantly reduced the number of NeuN+ cells compared to the saline group (*p* < 0.05, ANOVA-Tukey).^∗^Comparison of saline versus minocycline. ^+^Comparison of saline versus BMMCs. Arrows point to NeuN+ cells. ∗ demarcates lesion center (a–f). Scale bars: 400 *μ*m (a, c, e); 40 *μ*m (b, d, f).

Conspicuous neuronal loss was observed following ET-1-induced striatal ischemia (Figures 3(a) and 3(b)), which is in agreement with previous studies [7]. In this study, we comparatively assessed the effect of BMMC transplantation and minocycline treatment on neuronal preservation. Both experimental therapies reduced neuronal loss (Figures 3(c), 3(d), 3(e), and 3(f)). These results were confirmed by quantitative analysis (Figure 3(g)). The mean numbers of NeuN+ cells/field were 61.3 (±1.5), 86.8 (±3.4), and 81.0 (±3.4) for ischemic animals treated with sterile-saline, BMMCs, and minocycline, respectively (Figure 3(g)). The increased neuronal densities for the BMMC and minocycline groups compared to those for the saline group were statistically significant (^∗^*p* < 0.05, ANOVA-Tukey, Figure 3(g)). There was no statistical difference between the BMMC and minocycline groups (*p* > 0.05, ANOVA-Tukey, Figure 3(g)).

### 3.4. BMMC Transplantation Reduces More Apoptotic Cell Death than Minocycline following Striatal Ischemia

**Figure 4:**
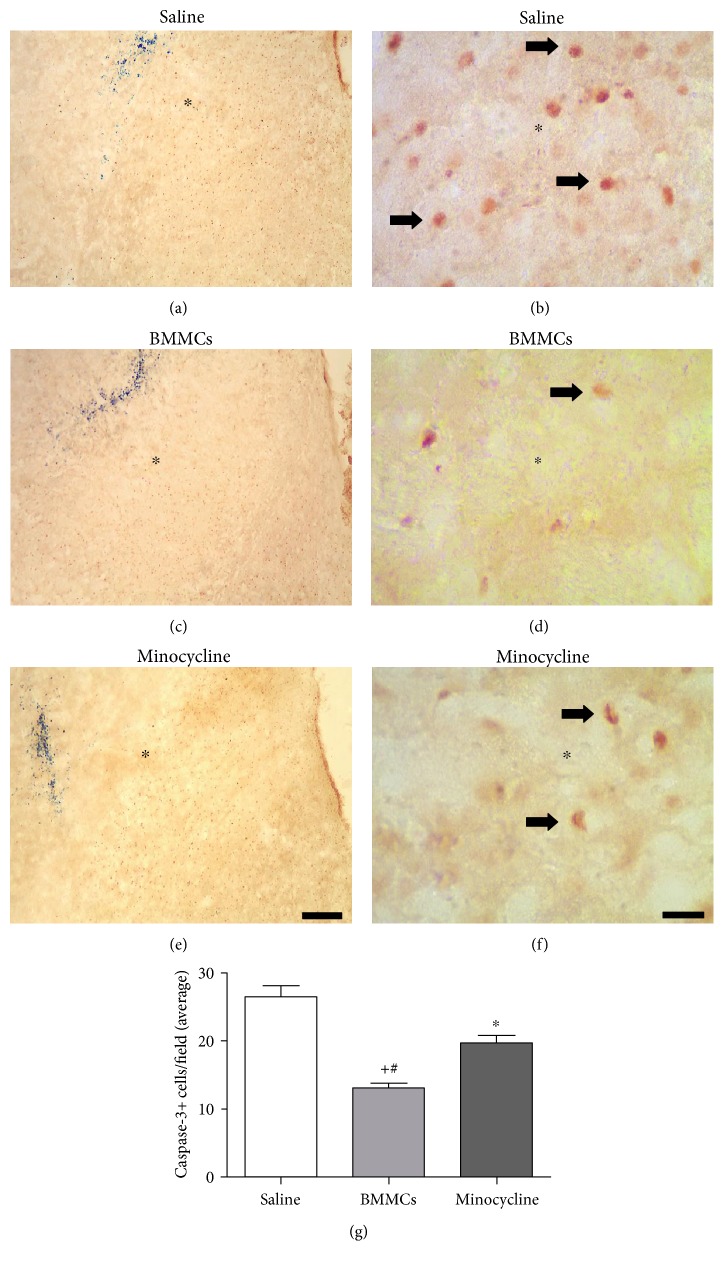
Antiapoptotic effect of BMMCs and minocycline treatment after striatal ischemia. Animals treated with sterile-saline (a, b), BMMCs (c, d), or minocycline (e, f) 7 days postischemic induction. Quantification of caspase-3+ cells in all experimental groups (g). BMMCs and minocycline treatment significantly reduced the number of caspase-3+ cells compared to the saline group (*p* < 0.05, ANOVA-Tukey), but BMMC effect was more significative compared to monocycline (*p* < 0.05, ANOVA-Tukey). ^∗^Comparison of saline versus minocycline. ^#^Comparison of saline versus BMMCs. ^+^Comparison of BMMCs versus minocycline. Arrows point to caspase-3+ cells. ∗ demarcates the lesion center (a–f). Scale bars: 400 *μ*m (a, c, e); 40 *μ*m (b, d, f).

Microinjections of ET-1 into the rat striatum induced apoptotic cell death as assessed by immunohistochemistry against active caspase-3+ cells (Figures 4(a) and 4(b)). We then compared the effects of BMMC transplantation and minocycline treatment on the number of caspase-3+ cells. Both BMMC transplantation (Figures 4(c) and 4(d)) and minocycline treatment (Figures 4(e) and 4(f)) decreased the number of caspase-3+ cells in the ischemic striatum, which was confirmed by quantitative analysis (Figure 4(g), *p* < 0.05, ANOVA-Tukey). The average numbers of caspase-3+ cells/field (±SD) were 26.5 (± 1.6), 13.1 (± 0.7), and 19.7 (±1.1) for ischemic animals treated with sterile saline, BMMCs and minocycline, respectively (Figure 4(g)). Reduced numbers of active caspase-3+ cells/field were statistically significant compared to those of the control group (Figure 4(g), ^∗^*p* < 0.05, ANOVA-Tukey). BMMC transplantation afforded a better reduction in the number of apoptotic cells in the ischemic striatum compared to the minocycline group (Figure 4(g), *p* < 0.05, ANOVA-Tukey).

### 3.5. BMMC Transplantation and Minocycline Treatment Induce Functional Recovery following Striatal Ischemia as Assessed by Specific Behavioral Tests

**Figure 5:**
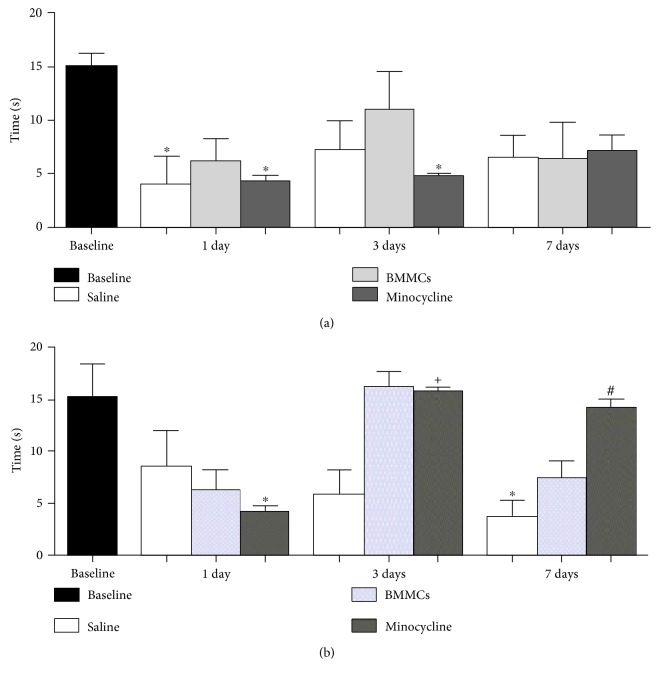
Comparison of sensorimotor behavior of the right and left paws at 1, 3, and 7 days after the ischemic event using stick-tape test. Right paw (a) and left paw (b). ^∗^Comparison with baseline. ^#^Comparison with the saline group. ^+^Comparison of BMMCs versus minocyline (*p* < 0.05, ANOVA-Tukey).

To assess the effects of BMMC transplantation and minocycline treatment on sensorimotor recovery following striatal ischemia, we used the modified sticky-tape [46] and open-field tests [45]. We first evaluated the performances of both right and left paws using sticky-tape test at 1, 3, and 7 days following striatal ischemia (Figure 5). For the right paw, saline- and minocycline-treated animals showed functional deficits at 1 day, while the BMMC group had no change (Figure 5(a)). On the third day, only the minocycline group differed from the baseline. Nevertheless, all groups showed similar sensorimotor behavior and no differences were observed compared to the baseline at 7 days (Figure 5(a)). For the left paw, the minocycline group showed functional deficit compared to the baseline at 1 day, but both the BMMC and minocycline groups did not show difference compared to the baseline at 3 and 7 days (Figure 5(b)). Only the saline group did not improve at 7 days compared to the baseline indicating functional recovery for both therapies.

**Figure 6:**
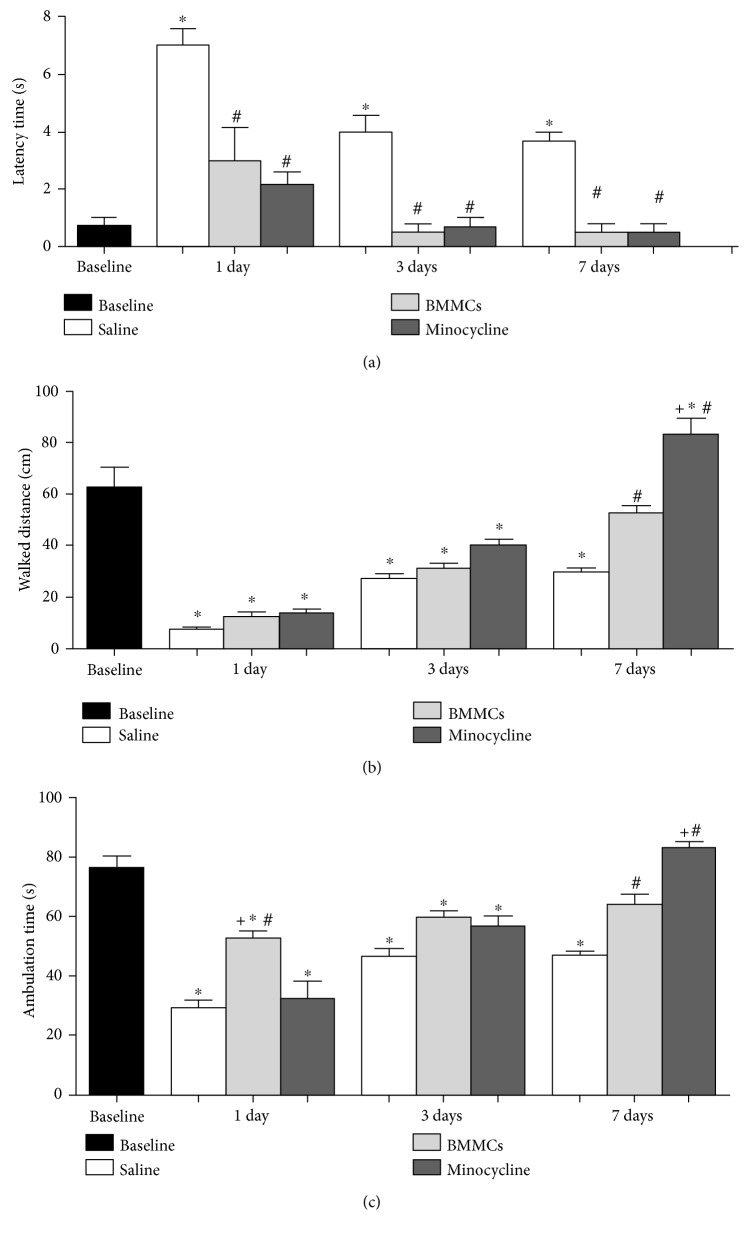
Open field analysis using latency, walked distance, and ambulation time parameters. Test results for latency (a), walked distance (b), and ambulation time (c). ^∗^Comparison with baseline. ^#^Comparison with the saline group. ^+^Comparison of BMMC versus minocyline (*p* < 0.05, ANOVA-Tukey).

**Figure 7:**
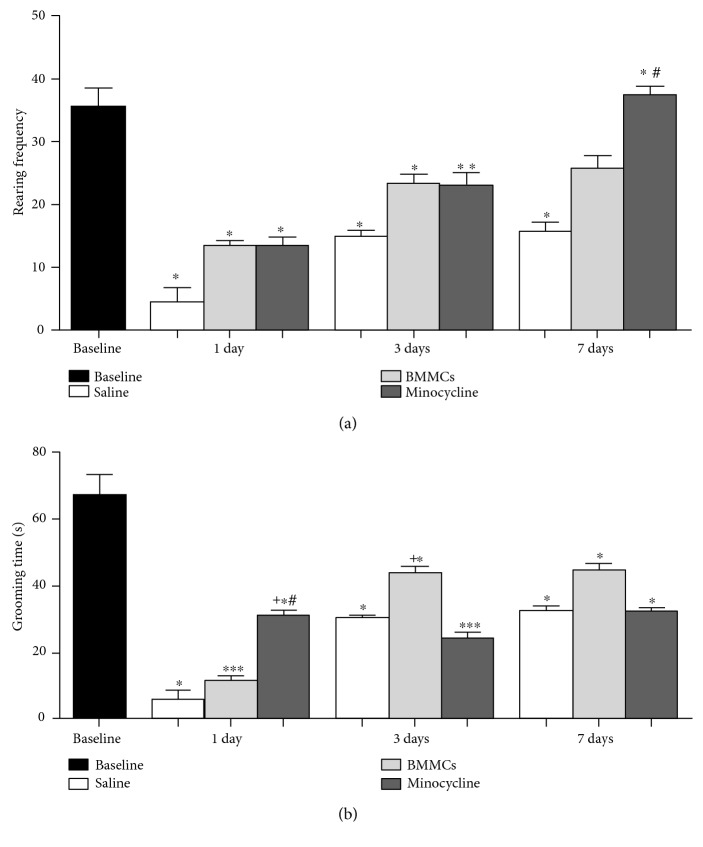
Open field analysis using rearing frequency and grooming time parameters. Test results for rearing frequency (a) and grooming time (b). ^∗^Comparison with baseline. ^#^Comparison with the saline group. ^+^Comparison of BMMC versus minocyline (*p* < 0.05, ANOVA-Tukey).

We then explored sensorimotor behavior using the open field test. We have evaluated the time that the animal took to start movement in the test box (latency). It was found that only saline-treated animals showed bradykinesia compared to the baseline. These animals showed increased latency in all investigated survival times (Figure 6(a)). Animals treated with minocycline or BMMCs showed no impairment in their ability to initiate movements in the test box (Figure 7(a)). Statistical comparisons between the BMMC and minocycline groups revealed differences compared to saline-treated animals, but not between themselves (Figure 6(a)). This has been observed for the other survival times (Figure 6(a)).

In order to quantify the explored space by the rat in the test apparatus, the walked distance was measured by counting the crossings in the text box lines (Figure 6(b)). In the first and third days, all groups showed decreased ambulation rate compared to the baseline (Figure 6(b), *p* < 0.05, ANOVA-Tukey). The BMMC and minocycline groups improved their ambulation rates by 7 days compared to the baseline but differed from animals treated with saline (Figure 6(b), *p* < 0.05, ANOVA-Tukey). In this survival time, animals treated with minocycline walked a longer distance than BMMC-treated animals (Figure 6(b), *p* < 0.05, ANOVA-Tukey).

The ambulation time was measured to all experimental groups (Figure 6(c)). All groups presented decreased ambulation time at 1 and 3 day survival times compared to the baseline (Figure 6(c)). At 7 days, BMMC and minocycline animals presented a longer ambulation time compared to saline-treated animals and did not differ from the baseline (Figure 6(c)). BMMC animals presented a longer ambulation time than saline-treated animals at 1 and 7 days, but minocycline-treated animals presented higher ambulation time only at 7 days post ischemia (Figure 6(c)).

We have also investigated the animal's behavior of standing on rear limbs in the test box and against the wall (rearing frequency). There was a decrease in the rearing frequency for all experimental groups after ischemia compared to the baseline at 1 and 3 days, but a slight improvement was observed in the later survival time (Figure 7). BMMC- and minocycline-treated animals had an improvement in function at 7 days, but minocycline-treated animals presented a better performance (Figure 7). Both BMMC- and minocycline-treated animals did not differ from the baseline at 7 days (Figure 7). Minocycline-treated animals presented an improvement compared to saline-treated animals, as well (Figure 7).

Finally, we have investigated the rat's self-cleaning behavior (grooming) (Figure 7(b)). The grooming time was lower in all experimental groups compared to the baseline (Figure 7(b)). Minocycline-treated animals presented higher grooming time compared to saline-treated animals or BMMC animals at 1 day (Figure 7(b)). Similar results were found at 3 days, except for the BMMC group, which presented a higher grooming time compared to saline- and minocycline-treated animals. There were no differences between groups at 7 days (Figure 7(b)).

## 4. Discussion

In this study, we have performed focal striatal ischemic damage by microinjections of ET-1 in order to comparatively investigate the effects of BMMC transplantation and minocycline treatment on the histopathological outcome and functional recovery. We addressed the patterns of ischemic lesion area, microglial activation, neuronal protection, programmed cell death, and functional recovery in both animals treated with saline and minocycline or transplanted with BMMCs. Both therapies induced comparable neuroprotection and functional recovery, but BMMC transplantation afforded better reduction of microglial activation and programmed cell death. Some functional recovery has been observed by both therapies with some peculiarities.

The ET-1 model of stroke has been validated by several groups including ours in both rodents [7, 15, 16, 35–37] and primates [40]. It is based on the vasoconstrictor effects of ET-1, which can be locally injected into the CNS to cause considerable reduction of the blood blow for about 3 hours [50]. ET-1 has been also used to occlude the middle cerebral artery in several experimental conditions [42, 50, 51]. One special particularity of ET-1 stroke model is its practicability. The surgical procedure is much simple compared to that of the MCAO filament model, another very used stroke model in rodents. In addition, ET-1 microinjections can be performed with very small amounts causing very focal ischemic damage, like in the capsular model of stroke [43].

To address the survival of transplanted BMMCs was not the purpose of this paper. Nevertheless, other authors investigated the survival of BMMCs or mesenchymal cells transplanted by intra-arterial injection following stroke [34]. The data showed that about 95% of BMMCs or mesenchymal cells intra-arterially transplanted after rat MCAO are shortly trapped in the spleen. At 6 hours, BMMCs are present in the brain parenchyma with a considerable increase at 12 hours. Their numbers start decreasing at 24 hours following MCAO. No BMMCs are present in the brain parenchyma at 2 weeks [34], which suggests a transient existence for these cells. Their neuroprotective and anti-inflammatory effects seem to occur in the acute phase of stroke [34].

The results showed that intrastriatal transplants of BMMCs induced a higher decrease in the number of ED1+ cells compared to animals treated with minocycline. It confirms and extends previous findings that BMMCs have a potent anti-inflammatory and immunomodulatory effect in experimental models of stroke [15, 16, 52, 53]. We have previously shown that intravenous BMMC transplantation induces significant neuroprotection and decreases microglia/macrophage activation [15, 16], but the present study adds new information to the field showing that the anti-inflammatory effect is higher than the one obtained by minocycline treatment. In addition, this study extended these previous results by modifying the route of transplantation. A comparative analysis of these experiments suggests that anti-inflammatory, neuroprotective, and functional recovery are more intense when BMMC transplantation is performed directly into the striatum. In these circumstances, more BMMCs have access to the brain parenchyma, considering that around 95% of BMMCs go to peripheral organs (like spleen) in the case of intravenous or intra-arterial transplantation [34]. Shortly, about 95% of BMMCs transplanted at 2 hours after MCAO are trapped in the rat spleen. They started to arrive into the brain at 6 hours and increased their numbers from 12 hours and starting to decrease from 24 hours after MCAO. At 2 weeks, no BMMCs were present in the brain parenchyma. These data show that BMMCs transplanted after stroke have a transient existence, and their effects seem to occur in the stroke acute phase.

Intrastriatal transplantation of BMMCs at 24 hours after ischemia reduced the lesion area in about 20% compared to intravenous transplantation performed in our previous studies [15]. This might be explained by an increased amount of BMMCs in the tissue and a better modulation of inflammatory response. Nevertheless, this may depend on transplantation time window, as later transplantation of BMMCs (7 days) is not so efficient in affording neuroprotection and anti-inflammatory effects [54].

Minocycline is a very effective inhibitor of microglia activation in experimental models of acute neural disorders in rodents [13, 55]. It seems that minocycline possesses pleiotropic actions, including mitochondrial protection, inhibition of caspases 1 and 3, metalloproteinase activity reduction, and microglial inhibition [56]. Tissue protection obtained by microglial inhibition is also related to decreased levels of TNF-*α* and other proinflammatory cytokines released by glial cells. Another important minocycline mechanism of action is inhibition of high-mobility group box 1 protein, which acts as a cytokine and is critical for inflammatory actions of microglial cells [57]. It is likely that the mechanisms of action of BMMCs are more efficacious and involve release of several anti-inflammatory cytokines with several downstream effects, including the one performed by minocycline.

Both minocycline and BMMCs considerably reduced the infarct area after experimental striatal stroke. These results confirm previous studies, which have shown that minocycline [13] or BMMCs [52, 58] greatly reduce the infarct area after MCAO. An interesting possibility is that concomitant treatment with minocycline and BMMCs has synergistic effects after stroke. Recent studies from our group suggested that concomitant treatment with minocycline and intravenous transplantation of BMMCs has synergistic effects after both cortical [15] and striatal [16] stroke induced by microinjections of ET-1. This possibility should be explored in further studies using intrastriatal transplants of BMMCs.

ET-1 microinjections induced massive neuronal loss after striatal ischemia. Both BMMC transplantation and minocycline treatment induced comparable neuroprotection, as revealed by preservation of NeuN+ cells. In our previous study, we found that treatment with minocycline preserves the white matter after striatal acute injury in adult rats induced by NMDA microinjection [55], which is in agreement with the results presented here. As previously suggested, further studies should investigate possible neuroprotective effects of these therapies applied concomitantly at different survival times.

The mechanisms by which both minocycline and BMMCS induce neuronal preservation are unknown. Nevertheless, the modulation of inflammatory response following stroke may be involved. Several studies suggest that modification of the pathological microenvironment can reduce neuronal loss and increases neuronal survival in the peri-infarct area. Hamby et al. showed that microglial inhibition with PJ34, an inhibitor of poly(ADP-ribose) polymerase, reduces the neuronal loss in the hippocampus up to 84% in an experimental model of global ischemia [17].

As previously suggested, similar mechanisms may be related to the neuroprotective actions of BMMCs. The anti-inflammatory effect of both therapies may be involved on the reported neuronal preservation. We have also described significant neuronal preservation after BMMC intravenous transplantation at 24 h following both cortical and striatal ischemia [15, 16]. The neuroprotection was increased after concomitant treatment with BMMCs and minocycline [15, 16] suggesting a synergic effect of concomitant treatment and that modulation of inflammation facilitates therapeutic actions of transplanted BMMCs. Similar approach may be used after intrastriatal transplantation of BMMCs.

BMMCs exhibit paracrine effects that may underlie the therapeutic benefits of BMMC therapy [59, 60]. Transplanted BMMCs can facilitate endogenous signals that increase neurovascular remodeling, contributing to endogenous neurogenesis after stroke [61]. The induction of neuroprotection by BMMCs may also be related to increased angiogenesis and arteriogenesis through the release of growth factors, proteases, and chemokines, such as transforming growth factor beta (TGF-*β*), basic fibroblast growth factor (FGFb), vascular endothelial growth factor (VEGF), and stromal-derived factor-1 (SDF1) [62–64].

Both minocycline treatment and BMMC transplantation reduced apoptotic cell death after striatal ischemia. Nevertheless, BMMC transplantation was more effective in reducing apoptotic cell death. In a recent study, we have reported that both therapies reduced the number of caspase-3+ cells after intravenous infusion of BMMCs after cortical ischemia [15]. In this study, Franco and colleagues have shown that concomitant treatment with BMMCs and minocycline more significantly decreased the number of apoptotic cells than the isolated treatments. In future studies, it should be investigated whether concomitant treatment with minocycline and intrastriatally transplanted BMMCs are also more effective in reducing apoptotic cell death.

Other authors reported that BMMC transplantation into organotypic hippocampal culture with oxygen and glucose deprivation also causes reduction in the number of caspase-3+ cells [65]. Intravenous transplantation of mesenchymal stem cells also reduces neuronal apoptosis after MCAO [66]. Release of trophic factors such as bFGF and VEGF seems to be responsible for the antiapoptotic effects of mesenchymal stem cells [67–69].

Several studies suggest that minocycline reduces apoptotic cell death after stroke [15, 16], excitotoxic injury [55, 70], and SCI [71, 72]. The primary antiapoptotic mechanism is the direct inhibition of cytochrome C release by mitochondria [73]. However, this effect seems to be dose dependent, as doses about or higher than 100 mg/kg may induce neuronal death [73]. The mechanisms by which BMMCs are more effective than minocycline in reducing apoptosis are unknown, but a more comprehensive pleiotropic effect of BMMCs by release of growth factor may be involved.

The use of specific behavioral tests revealed that both minocycline treatment and BMMC transplantation improved sensorimotor performance of ischemic animals compared to control. These results are consistent with recent studies from our group, which used intravenous transplantation of BMMCs after ischemic damage to motor cortex [15] and striatum [16]. Other authors reported, separately, the effects of minocycline [41, 74] or BMMCs [75, 76] on functional recovery of ischemic animals, but no studies have compared the effects of these experimental therapies after striatal stroke and with intravenous transplantation of BMMCs.

It is noteworthy that the evaluation of sensory motor activity of the upper limbs is imperatively made because this motor behavior is essential for the performance of routine activities, which should be prioritized in the evaluation of stroke endpoints. The results of this study show functional recovery of the upper body quadrant. This is clinically important, as 85% of the patients who had stroke, around 55 to 75% may present functional deficit in the arms even three months after stroke onset if they do not receive proper treatment [77, 78]. It follows that future studies using minocycline and BMMC transplantation in humans may establish a promising therapy to minimize damage and maximize functional recovery after stroke.

Animals treated with BMMCs and minocycline did not show bradykinesia as compared to animals that received only sterile saline solution. This suggests that these therapies are promising approaches to ensure functional stability in motion initiation capability. In patients with ischemic brain injury, motor deficits directly impact on functional capacity, socialization, and life's quality, which impairs rehabilitation as well as social interaction [79–81].

In addition, it is necessary to consider the patient's autonomy during their daily activities after stroke. Therefore, the execution of semi-independent or independent normal tasks configures an important criterion for this patient's life quality. Based on this, this research investigated the behavior of standing up, self-cleaning, and ambulation in the open field exploratory test. The results showed that the ischemic animals treated with saline solution had worse performance than those treated with BMMCs and minocycline. These findings have significant clinical and functional importance, considering that motor and sensory deficits may affect the patient's daily living and self-care activity.

## 5. Conclusion

Microinjections of 80 pmol of ET-1 in adult rats' striatum produce focal ischemia with a clear infarct area, intense microglia activation, apoptosis, and sensorimotor impairment. Minocycline treatment or BMMC transplantation reduces the infarct area, inhibits microglial activation, reduces apoptosis, and induces functional recovery. Comparatively, BMMC transplantation affords a more intense microglial inhibition and reduction of apoptosis than minocycline. Nevertheless, both experimental therapies induce a similar degree of neuronal preservation and functional recovery. The results suggest that both minocycline and BMMC transplantation are promising therapeutic approach to minimize damage and maximize functional recovery after stroke. Future studies using longer survival times are required to investigate whether the observed effects are long lasting.
